# Distinct Nodal Centrality and Connectivity Profiles Underlying Independent and Interdependent Self-Construal: A Graph-Theoretical Resting-State fMRI Study

**DOI:** 10.3390/brainsci16101021

**Published:** 2026-09-25

**Authors:** Zilun Xiao, Xiong Xiao, Jian Lu, Zhuoya Yang, Ying Jia, Yingcan Zheng

**Affiliations:** 1 School of Innovation and Entrepreneurship Education, Chongqing University of Posts and Telecommunications, Chongqing 400065, China; xiaozl@cqupt.edu.cn (Z.X.); jiaying@cqupt.edu.cn (Y.J.); 2 Department of Psychology, Southwest University, Chongqing 400715, China; s230501014@stu.cqupt.edu.cn; 3 College of Teacher Education, Xichang University, Xichang 615000, China; xcc20170038@xcc.edu.cn; 4 Department of Basic Psychology, School of Medical Psychology, Army Medical University, Chongqing 400038, China; yangzhuoya@tmmu.edu.cn; 5 Developmental Psychology for Armyman, Department of Medical Psychology, Army Medical University, Chongqing 400038, China

**Keywords:** self-construal, resting-state fMRI, graph theory, degree centrality, functional connectivity, in-group favoritism

## Abstract

**Highlights:**

**What are the main findings?**

- Independent self-construal is associated with degree centrality in occipital perceptual hubs (left inferior occipital gyrus, right lingual gyrus) and, in exploratory analyses, with more variable dynamic coupling to frontoparietal control regions.
- Interdependent self-construal is associated with degree centrality in the medial prefrontal cortex and thalamus; a relatively more independent profile predicts stronger in-group favoritism in a minimal-group setting.

**What are the implications of the main findings?**

- Trait self-construal is associated with the nodal centrality of perceptual and social-integrative hubs and with their resting-state connectivity.
- These findings connect self-construal with intrinsic hub organization and identify connectivity correlates of in-group favoritism.

**Abstract:**

Background/Objectives: Self-construal—the extent to which the self is construed as autonomous from or connected with others—shapes cognition and social behavior, yet its system-level neural architecture remains poorly understood. This study examined whether independent and interdependent self-construal are associated with distinct patterns of nodal centrality and connectivity in the resting brain and whether these patterns covary with in-group favoritism. Methods: Graph-theoretical analyses were applied to resting-state fMRI data from 67 college students. Degree centrality (DC) was the primary analysis; seed-based static functional connectivity (sFC) and dynamic functional connectivity variability (FCV) from DC-derived seeds served as exploratory follow-ups. In-group favoritism was assessed with a minimal-group point-allocation task. Results: Independent self-construal was positively associated with DC in the left inferior occipital gyrus and right lingual gyrus; exploratory sFC linked these occipital seeds to frontal, temporal, and parietal regions, with greater exploratory FCV in their coupling with frontoparietal control areas. Interdependent self-construal was positively associated with DC in the medial prefrontal cortex and thalamus, with exploratory sFC to occipital, temporal, and insular regions but no significant FCV effects. The self-construal difference score yielded convergent DC results and was negatively associated with in-group favoritism; occipital–postcentral coupling was positively associated with in-group favoritism in an exploratory correlation. Conclusions: Independent and interdependent self-construal are associated with distinct nodal centrality profiles, linking independence to occipital perceptual hubs and interdependence to medial prefrontal–thalamic hubs. Exploratory connectivity analyses extend these findings to the coupling of these hubs with distributed brain regions and to in-group favoritism.

## 1. Introduction

Self-construal describes how individuals perceive their relationships with themselves and others, fundamentally shaping cognition and behavior. Markus and Kitayama’s [1] framework remains influential: independent self-construal centers on autonomy and separateness (common in individualistic societies), while interdependent self-construal emphasizes social harmony and connection (central to collectivistic cultures) [1,2]. These two construals support different cognitive strategies, from self-focused attention to other-oriented sensitivity. A basic question follows: how does the brain’s intrinsic functional architecture support these contrasting self-views?

Regional brain activity differs with self-construal. Meta-analyses show that interdependent individuals exhibit greater activation in the dorsal MPFC, lateral PFC, and TPJ during socio-emotional tasks. Conversely, independent individuals show stronger activation in ACC, ventral MPFC, bilateral insula, and right temporal pole [3]. The mPFC—involved in both self and other processing—is particularly sensitive to self-construal differences [4,5]. Structural studies point the same way: independent self-construal links to increased grey matter volume in frontal (ventral MPFC, DLPFC) and parietal–occipital areas [6]. Together, these studies show distinct neural patterns for each self-construal type. Recent evidence further indicates that self-construal modulates functional connectivity within the default mode network [7] and that cultural differences in self-construal are associated with distinct patterns of parahippocampal gyrus and prefrontal connectivity [8].

Functional connectivity studies extend this picture. A machine learning study found that independent self-construal relates more to right-hemisphere connectivity, whereas interdependent self-construal relates more to the left [9]. Seed-based analyses add detail: independent self-construal shows positive connectivity within DMN (PCC/PCU, MPFC) and ECN (TPJ). Notably, relatively stronger independent (versus interdependent) self-construal is associated with stronger negative connectivity between the DMN and ECN [10]. These findings motivate examining how the centrality of individual brain regions within whole-brain functional networks relates to self-construal.

Still, an important gap remains: how self-construal relates to the nodal centrality of regions across the whole brain. Regional activation analyses characterize local responses, whereas seed-based connectivity examines connections with selected regions. Graph theory complements these approaches by modeling the brain as nodes (regions or voxels) connected by edges (rs-fMRI correlations) and quantifying the connectedness of each node [11,12,13]. In particular, degree centrality can identify highly connected hub regions within whole-brain functional networks [14]. Mapping these hub properties can therefore clarify how intrinsic functional organization supports different self-views.

The present study addresses this gap. We collected self-construal measures and resting-state functional MRI (rs-fMRI) data from 67 participants to test whether independent and interdependent self-construal exhibit different patterns of nodal centrality and connectivity. Building on evidence of hemispheric asymmetries [9] and network segregation [10], we expected the two dimensions to show different profiles of nodal centrality and connectivity. Specifically, we hypothesized that independent self-construal would be associated with hub properties of regions supporting self-referential and executive processing. Interdependent self-construal, in contrast, would engage regions supporting social-contextual integration, including the medial prefrontal cortex (MPFC), a core substrate of self-referential processing [15], and social-cognitive hubs such as the temporoparietal junction [16]. We used a whole-brain, data-driven DC analysis to identify self-construal-related hubs without restricting the search to these candidate regions. Degree centrality served as the primary nodal analysis, with static and dynamic connectivity as exploratory follow-ups. Throughout, we use network organization in the specific sense of nodal hub structure (degree centrality); global topological properties such as modularity or small-worldness were not assessed. This design links the centrality of self-construal-related hubs to their connectivity profiles.

## 2. Methods

### 2.1. Participants

Seventy college students (15 men, 55 women; mean age = 20.59 years, SD = 1.80) were recruited. To ensure the successful completion of the MRI scan, all participants were confirmed to have no major physical illnesses, normal or corrected vision, no metallic implants, and no history of substance addiction or mental illness. The experimenters explained the precautions for MRI scanning, and all participants provided written informed consent. Upon completion of the experiment, all participants were compensated with RMB 70. The study protocol was approved by the Ethics Committee of Southwest University (protocol code H20079 and 31 December 2020) prior to its commencement.

### 2.2. Measures

#### 2.2.1. Self-Construal

After the resting-state scan, participants completed the Chinese version of the Self-Construal Scale (SCS) [17], translated and revised by Wang et al. [18], in an isolated room. The scale comprises two 12-item subscales—independent and interdependent self-construal—rated on a 7-point Likert scale (1 = strongly disagree, 7 = strongly agree). Sample independent items include “My personal identity, independent of others, is very important to me,” and sample interdependent items include “I will sacrifice my self-interest for the benefit of the group I am in.” In the present sample, Cronbach’s α was 0.73 for the independent subscale and 0.77 for the interdependent subscale.

#### 2.2.2. In-Group Favoritism

In-group favoritism was assessed with a point-allocation (MATRIX) task. Participants allocated points between an anonymous in-group member and an anonymous out-group member, identified by team color and code number, in a minimal-group setting. They were informed that the allocated points were related to final rewards and that their own payoff would likewise be determined by another participant’s allocation. In-group favoritism was quantified as the difference between the total points allocated to the in-group member and those allocated to the out-group member (difference score, DS), with DS > 0 indicating in-group favoritism, DS < 0 indicating out-group favoritism, and DS = 0 indicating fairness.

### 2.3. MRI Data Acquisition

MRI scanning was performed on a Siemens MAGNETOM Prisma_fit 3.0 T scanner (Siemens Healthineers, Erlangen, Germany; software version syngo MR E11) with a 64-channel head–neck coil. A total of 240 echo-planar imaging (EPI) volumes were collected. Parameters of the rs-fMRI scanning were as follows: TR = 2000 ms; TE = 30 ms; number of slices = 62; flip angle (FA) = 90°; field of view (FOV) = 224 × 224 mm^2^; matrix size = 112 × 112; slice thickness = 2 mm; slice gap = 0.3 mm; voxel size = 2 × 2 × 2 mm^3^; multiband (simultaneous multi-slice) acceleration factor = 2; in-plane GRAPPA acceleration factor = 2. High-resolution T1-weighted anatomical images were acquired using a sagittal magnetization-prepared rapid gradient-echo (MPRAGE) sequence (TR = 2530 ms; TE = 2.98 ms; inversion time = 1100 ms; FA = 7°; FOV = 224 × 256 mm^2^; 192 sagittal slices; slice thickness = 1 mm; voxel size = 1 × 1 × 1 mm^3^, reconstructed at 0.5 × 0.5 mm in-plane).

### 2.4. MRI Data Preprocessing

DPABI (http://rfmri.org/dpabi/, accessed on 22 July 2022 ) [19] and SPM12 (https://www.fil.ion.ucl.ac.uk/spm/, accessed on 22 July 2022) toolboxes in MATLAB-R2018a (MathWorks, Natick, MA, USA) were applied to preprocess the MRI data. The first 10 volumes of functional images were discarded. Then, slice timing correction (performed in SPM12 using the per-slice acquisition times of the multiband sequence) and realignment were performed. The functional images were then reoriented to the anterior commissure. T1 images were co-registered to functional images and segmented into grey matter, white matter (WM) and cerebrospinal fluid (CSF). The DARTEL algorithm was applied to create a study-specific template for normalization [20]. The Friston 24-parameter model [21] was applied to regress out head motion parameters. Combined with participant-level FD screening and the group-level mean-FD covariate, this approach follows current recommendations for limiting motion-related bias in functional connectivity studies [22,23]. The first five principal components of the WM and CSF signals were also regressed out as covariates. Global signal regression was not applied in the reported analyses. This choice preserves global components that may contain neural signal, while motion and WM/CSF regressors account for known nuisance sources. GSR alters the correlation distribution and can introduce negative correlations, and its use depends on the scientific question and analysis framework [24,25]. Head-motion quality control was based on the percentage of volumes with framewise displacement (FD) exceeding 0.2 mm; three participants (sub24, sub40, and sub44) were excluded because more than 20% of their volumes showed FD exceeding 0.2 mm, yielding a final sample of 67 participants for all subsequent analyses. The functional images were then normalized into MNI space using the DARTEL-generated template and resampled to 2 × 2 × 2 mm^3^ voxel size. Spatial smoothing with a 4 mm FWHM Gaussian kernel was then applied, followed by a bandpass filter (0.01–0.1 Hz). For the DC analysis, computation was performed on unsmoothed data, and the resulting DC maps were smoothed afterwards with the same kernel.

### 2.5. Degree Centrality (DC) Analysis

DC is defined as the temporal association between a voxel and all other voxels of the brain within the grey matter mask. An n × n matrix of Pearson’s correlation coefficients was obtained (*n* = voxel number), with a threshold set at *r* > 0.25 [26,27]. This threshold was selected a priori to exclude weak correlations, following prior voxel-wise hub mapping [26]. Weighted positive DC was calculated as the sum of the retained correlation weights for each voxel, with correlations at or below the threshold excluded [13]. Buckner et al. [26] found that cortical hub topography was qualitatively similar across the thresholds they examined, providing methodological support for this choice. Each participant’s DC map was then divided by its whole-brain mean to obtain the mean-normalized DC map (mDC). Finally, all maps were smoothed with a 4 mm FWHM Gaussian kernel.

### 2.6. Static Functional Connectivity (sFC) Analysis

DPABI was used to conduct the sFC analysis. Spatial smoothing with a 4 mm FWHM Gaussian kernel was performed before FC analysis. A seed-based FC analysis was used as an exploratory follow-up. Significant clusters from the primary DC analyses were defined as regions of interest (ROIs) and used as seeds for the subsequent exploratory sFC and dFC/FCV analyses. Because the seed ROIs were defined in the same sample, these follow-up analyses are exploratory and serve to characterize the connectivity of the identified hubs. The time course of each ROI was then extracted and correlated with the time series of each voxel within the grey matter mask to obtain FC maps. Finally, Fisher’s z transformation was applied to all FC maps to improve normality.

### 2.7. Dynamic Functional Connectivity (dFC) Analysis

The DynamicBC toolbox [28] was used to perform dFC analysis. The sliding window approach was applied. Data were split into 60 s (30 TR) tapered windows (convolved with a Gaussian kernel), with a step of 2 s (1 TR) progressively shifted from the onset of the previous window, resulting in a total of 201 windows. Pearson’s correlations between time series of each ROI and all other voxels within each window were calculated. The FC variability (FCV) map was then formed by computing the standard deviation (SD) of these correlation coefficients (after Fisher’s z-transformation per window) across all windows within each voxel. No additional Fisher’s z transformation was applied to the FCV maps because FCV is a standard-deviation measure rather than a correlation coefficient; FCV maps were entered into group-level inference without this transformation. The window length of 30 TR (60 s) was chosen based on prior work suggesting that this range balances the trade-off between capturing reliable connectivity estimates and detecting dynamic fluctuations [29,30]. Throughout, dFC refers to the sliding-window analysis framework, whereas FCV—the standard deviation of windowed correlations—is the actual per-voxel measure entered into the group-level analyses.

### 2.8. Statistical Analysis

Correlation analyses were conducted between self-construal (independent or interdependent) and imaging maps (DC, sFC, FCV) using SPM12. The voxelwise DC analyses were specified as primary, whereas the seed-based sFC and FCV analyses were exploratory follow-ups. First, clusters showing significant DC associations with self-construal were identified by correlating self-construal scores with the DC maps, controlling for age, sex, and mean FD; these clusters were then defined as ROIs for the subsequent exploratory sFC and dFC/FCV analyses. Then, correlations between self-construal scores and sFC maps/FCV maps were calculated with age, sex and mean FD as covariates. The primary voxel-wise models examined the two dimensions separately; complementary SC-diff models examined their relative dominance. The cluster-level sensitivity analysis described below additionally adjusted for the other self-construal dimension. The statistical significance was set to an uncorrected voxel-wise threshold of *p* < 0.001 combined with a cluster-level family-wise error (FWE) corrected threshold of *p* < 0.05 for all voxel-wise imaging analyses. Statistical maps were visualized with the BrainNet Viewer [31]. As a sensitivity check, the mean mDC value of each significant cluster was extracted for every participant, and partial correlations between each self-construal dimension and its associated clusters were computed while controlling for the other dimension, age, sex, and mean FD (see Figure 1).

To link the neural findings to group-level behavior, Pearson correlations were computed between the extracted FC values of significant links and DS.

## 3. Results

### 3.1. Behavioral Results

Of the 67 participants included in the final analysis (15 males, 52 females; mean age = 20.57 years, SD = 1.86), independent self-construal scores ranged from 43 to 78 (M = 56.37, SD = 7.68), and interdependent scores ranged from 42 to 81 (M = 61.42, SD = 7.00). The two dimensions were weakly correlated, r(65) = 0.24, *p* = 0.0504. Neither dimension was significantly correlated with age (independent: r = −0.04, *p* = 0.732; interdependent: r = 0.10, *p* = 0.425). Males scored significantly higher than females on independent self-construal (t(65) = 3.01, *p* = 0.004; males: M = 61.33, SD = 8.23; females: M = 54.94, SD = 6.96), but not on interdependent self-construal (t(65) < 1, *p* = 0.99; males: M = 61.40, SD = 6.63; females: M = 61.42, SD = 7.17). Following previous work [32,33], a self-construal difference score (interdependent minus independent; hereafter SC-diff) was computed to index the relative dominance of the two dimensions. On the MATRIX task, the in-group favoritism difference score (DS; M = 22.91, SD = 29.90, range = −18 to 102) was negatively correlated with SC-diff, r(65) = −0.26, *p* = 0.034, indicating that a relatively more independent self-construal was associated with stronger minimal-group in-group favoritism; neither subscale alone was significantly correlated with DS (independent: r = 0.11, *p* = 0.389; interdependent: r = −0.21, *p* = 0.088).

### 3.2. DC Results

Correlation analyses revealed significant positive relationships between independent scores and DC values in the left inferior occipital gyrus (IOG; k = 109; peak MNI: −22, −88, −6; Z = 4.61) and the right lingual gyrus (k = 128; peak: 26, −84, −8; Z = 4.33). Interdependent scores were positively associated with DC in the ventral medial prefrontal cortex (gyrus rectus; k = 211; peak: 6, 18, −20; Z = 5.17) and the left medial pulvinar thalamus (k = 106; peak: −14, −28, 12; Z = 4.74) (Table 1). All reported clusters survived cluster-level FWE correction (*p* < 0.05) at a voxel-wise threshold of *p* < 0.001. These findings are illustrated in Figure 2. Scatter plots of the individual data are shown in Figure 3. Sensitivity analyses on the extracted cluster-mean DC values showed that all four associations remained significant after controlling for the other self-construal dimension, age, sex, and mean FD (partial r = 0.54 for the left IOG, 0.50 for the right lingual gyrus, 0.60 for the gyrus rectus/ventral MPFC, and 0.54 for the left medial pulvinar thalamus; all *p* < 0.001), showing that the positive associations in these clusters persisted after adjustment for the other dimension.

Complementary analyses using the difference score (SC-diff) converged with the dimension-wise results. SC-diff was positively correlated with DC in the ventral medial prefrontal cortex (gyrus rectus; peak MNI: 6, 18, −20; k = 90; *Z* = 4.02; cluster-level *p*(FWE) = 0.016) and negatively correlated with DC in the left inferior occipital gyrus (peak: −22, −88, −4; k = 271; *Z* = 5.03; *p*(FWE) < 0.001) and the right lingual gyrus (peak: 26, −84, −8; k = 206; *Z* = 4.83; *p*(FWE) < 0.001) (Table 2).

### 3.3. Exploratory sFC Results

Independent self-construal was positively associated with exploratory sFC from the left inferior occipital gyrus and right lingual gyrus seeds to frontal, temporal, parietal, and occipital regions. Interdependent self-construal was positively associated with exploratory sFC from the right gyrus rectus/ventral MPFC to the left lingual and right calcarine regions, and from the left medial pulvinar thalamus to inferior temporal, calcarine, middle occipital, fusiform, and insular regions. Results of the dimension-wise seed analyses are presented in Table 3, and those of the SC-diff-based seed analyses in Table 4.

Exploratory convergent results were obtained with SC-diff: SC-diff was negatively correlated with FC between the left IOG seed and the right lingual gyrus (k = 459; peak: 10, −90, −10; *Z* = 4.73) and the left postcentral/parietal region (k = 441; peak: −16, −30, 74; *Z* = 4.05), and with FC between the right lingual seed and the bilateral occipital cortex (k = 1600; peak: −18, −66, −12), the right lingual gyrus (k = 312; peak: 8, −56, −4), the right calcarine cortex (k = 113; peak: 20, −82, 16), the left superior frontal gyrus (k = 68; peak: −16, 64, 8), the bilateral postcentral gyri (k = 491 and k = 150), and the right precuneus (k = 84; peak: 6, −34, 58) (all cluster-level *p*(FWE) < 0.05; Table 4). Descriptively, these associations indicate that a relatively more independent self-construal was associated with stronger occipital-centered connectivity.

### 3.4. Exploratory Functional Connectivity Variability (FCV) Results

In the exploratory FCV analysis, independent self-construal scores were positively correlated with FCV between the left IOG and the right middle frontal gyrus (k = 89; peak MNI: 38, 36, 32; Z = 4.57), the right precentral gyrus (k = 101; peak: 52, 12, 36; Z = 4.34), and the right inferior parietal lobule (k = 40; peak: 44, −48, 54; Z = 4.26). Independent scores were also related to FCV between the right lingual gyrus and the left superior frontal gyrus (k = 35 and k = 31; peaks: −28, 38, 36 and −16, 22, 62), the right precentral gyrus (k = 30; peak: 52, 6, 36), the right superior parietal lobule (k = 48; peak: 20, −66, 48), and the right middle temporal gyrus (k = 26; peak: 50, −56, 2) (Table 5; see also Figure 4). No significant relationship was detected between interdependent scores and FCV measures at the same threshold.

### 3.5. Exploratory Brain–Behavior Correlations

In a secondary exploratory analysis, the FC values of the significant occipital–postcentral links were extracted and correlated with DS. DS was positively correlated with FC between the left IOG and the left postcentral gyrus (*r* = 0.26, *p* = 0.034) and with FC between the right lingual gyrus and the right postcentral gyrus (*r* = 0.29, *p* = 0.017; *N* = 67), suggesting that stronger occipital–postcentral coupling is associated with greater in-group favoritism. These brain–behavior correlations were exploratory and were not corrected for multiple comparisons.

## 4. Discussion

We treated voxelwise degree centrality (DC) as the primary analysis and used DC-derived seeds for exploratory static functional connectivity (sFC) and functional connectivity variability (FCV) follow-ups in 67 participants. Independent self-construal scores were positively correlated with DC values in the left inferior occipital gyrus and right lingual gyrus, whereas interdependent scores were positively correlated with DC values in the medial prefrontal cortex (MPFC) and thalamus. Exploratory sFC and FCV analyses from these same-sample DC-derived seeds showed additional associations with self-construal. No significant relationships were found between interdependent scores and FCV measures. The four DC associations also persisted in the cluster-level sensitivity analysis controlling for the other self-construal dimension, age, sex, and mean FD. Overall, the results relate self-construal to the centrality of perceptual and social-integrative hubs and describe how these hubs couple with the wider brain.

The occipital centrality findings extend earlier task-based and seed-based accounts of self-construal [9,10]. Task-evoked activation identifies regions recruited during particular cognitive operations, whereas resting-state DC measures a voxel’s aggregate functional connectedness with the rest of the brain. Evidence that self-construal priming modulates early occipital responses during global/local perception [34] provides a basis for linking the present occipital findings to perceptual processing. The DC results therefore add a complementary perspective: individual differences in self-construal are associated with the intrinsic connectedness of perceptual hubs, alongside the medial prefrontal and thalamic hubs associated with interdependence.

### 4.1. The Neural Substrates Underlying Independent Self-Construal

Our results link two occipital regions, the left inferior occipital gyrus and the right lingual gyrus, to independent self-construal across multiple connectivity measures (DC, sFC, and FCV). The IOG is best known for visual processing, object recognition, and face perception [35,36]. It has also been implicated in interpersonal perception and social visual processing [37,38]. The core difference between independent and interdependent self-construal lies in how individuals perceive themselves relative to others [1]. Individual differences in this visual-social hub may therefore contribute to independent self-views. Consistent with this possibility, independent self-construal modulates early visual processing in the extrastriate cortex by facilitating local over global feature detection [34]. Independent self-construal emphasizes the separation of the self from the social context and prioritizes unique personal attributes [1]. This analytical, feature-oriented visual style may be supported by inferior occipital regions, which are well suited to fine-grained visual analysis and object-based attention [35]. The IOG-centered connectivity observed in more independent individuals may thus reflect a trait-like tuning toward fine-grained discrimination of environmental and social signals.

The right lingual seed also showed stronger connectivity with higher-order regions, including the parahippocampal gyrus, fusiform gyrus, and angular gyrus. These areas support memory, face recognition, and semantic processing [39,40,41]. These functions accord with the trait-focused, self-expressive tendencies of independent self-construal. The integrative-self account proposes that self-reference binds perceptual and memory representations and coordinates them with attention [42]. This account provides a theoretical basis for interpreting the lingual-seed connectivity pattern as a link between visual representations and higher-order processes relevant to self-construal.

The exploratory FCV results further link independent self-construal to variable coupling between the IOG and frontoparietal regions. Task studies show that frontoparietal hubs alter their connectivity across cognitive demands [43], and that more integrated dynamic network states accompany faster and more accurate performance [44]. These findings offer a functional context for interpreting temporal variation in perceptual–control coupling [30,45]. The frontal and parietal regions in our FCV results, including the middle frontal gyrus, superior frontal gyrus, superior parietal lobule, and inferior parietal lobule, are key nodes of the frontoparietal control network. This network supports adaptive operations such as task switching, attentional reallocation, and goal-directed planning [43,46]. The enhanced variability in IOG–frontoparietal coupling may therefore support flexible allocation of attentional resources, a capacity that aligns with the context-independent, self-guided behavioral profile of independent self-construal [1,47]. A consistent self-guided orientation can coexist with flexible network coordination in the service of internally generated goals [43,48]. This interpretation converges with behavioral work linking independent self-construal to greater cognitive flexibility in context-independent reasoning [47,49]. The present resting-state FCV associations are consistent with this account of perceptual–control coordination.

### 4.2. The Neural Substrates Underlying Interdependent Self-Construal

For interdependent self-construal, two regions stood out: the MPFC and the thalamus. The MPFC is a well-established hub for self-referential processing, social cognition, mentalizing, and emotional regulation [50,51]. Its heightened degree centrality in interdependent individuals suggests that their self-referential processing is preferentially embedded in social contexts. This pattern accords with the interdependent emphasis on relational connectedness and group harmony. The thalamus, beyond its classic role as a sensory relay, actively participates in cortico-cortical communication and cognitive integration [52]. It maintains dense connections with the MPFC and supports the updating of self-related representations [53]. Cross-species evidence further shows that social cues from multiple sensory modalities converge in thalamic subregions, which then modulate social behavior through widespread cortical projections [54]. For individuals high in interdependent self-construal, continuous attunement to social norms and relational contexts is a core cognitive demand. Elevated thalamic degree centrality may therefore reflect an enhanced capacity to integrate multimodal social information. Through reciprocal connections with the MPFC, this integrative function may support socially embedded self-referential processing and allow flexible adjustment of self-representation to interpersonal contexts [55].

The MPFC seed showed stronger sFC with occipital regions, including the lingual gyrus and calcarine cortex. One likely account is top-down modulation: social and self-referential regions may tune early visual areas, rather than occipital regions carrying out social processing themselves. Such coupling could make interdependent individuals more sensitive to socially relevant visual input. This interpretation is consistent with evidence that the ventral MPFC is engaged when people judge relational self-worth, a construct central to interdependent self-construal [56,57]. From this perspective, the fronto-occipital circuit biases early visual processing toward social cues, helping interdependent individuals pick up information about social relationships [58]. The thalamus seed showed a similar pattern, with stronger sFC to the inferior temporal gyrus, fusiform gyrus, occipital cortex, and insula. This is consistent with the thalamus acting as an integrative hub rather than a simple relay [59]. Pulvinar pathways, in particular, prioritize socially salient visual signals such as faces and emotional expressions [60]. The thalamic–insula link may add interoceptive information to this process, which matters for interpersonal harmony and emotional attunement [61].

For interdependent self-construal, the detected associations involved DC and sFC; no FCV association reached the prespecified threshold. Thus, the present results characterize interdependence primarily through hub centrality and static coupling.

In summary, the two self-construal dimensions showed distinct centrality and connectivity patterns. Independent self-construal was tied to a visual network centered on the left IOG and right lingual gyrus, and its coupling with frontoparietal control regions was more variable. This profile may allow flexible, internally guided shifts in attention and perception. Interdependent self-construal, by contrast, was tied to the MPFC and thalamus, with stronger static connectivity to visual and insular regions. This profile fits social-contextual processing and multimodal integration. Taken together, the findings suggest that self-construal is reflected in the hub organization of both perceptual and social-integrative systems.

The direction of the brain–behavior association also deserves attention. In-group favoritism is often intuitively linked to interdependent orientations, but we found the opposite: a relatively more independent self-construal was associated with stronger minimal-group in-group favoritism. This direction is consistent with Nakashima et al. [33], who reported stronger in-group favoritism among individuals with independent self-construal, particularly under self-esteem threat. In minimal-group settings, group membership carries no established relational meaning. Favoring the in-group may therefore serve self-enhancement rather than affiliative motives, which accords with the self-focused regulatory style of independent self-construal. Within this framework, the occipital–postcentral coupling may covary with a perceptual–sensorimotor pattern relevant to allocative behavior.

The exploratory brain–behavior correlations link stronger occipital–postcentral coupling to greater in-group favoritism, complementing the association between SC-diff and allocation behavior. These are correlational links.

### 4.3. Limitations

Several limitations should be noted. First, our sample exhibited a marked sex imbalance (15 males and 55 females in the recruited sample; 15 males and 52 females in the final sample), and males scored significantly higher than females on independent self-construal (see Behavioral results). Given well-documented sex differences in self-construal [62,63], this imbalance may limit the generalizability of our findings and may obscure sex-specific connectivity patterns; future studies with balanced samples are needed to dissociate sex effects from self-construal effects. Second, the primary voxel-wise models analyzed the dimensions separately. The cluster-level sensitivity analysis retained all four positive DC associations after controlling for the other dimension and covariates (Section 3.2), while whole-brain simultaneous-entry models would further characterize their unique spatial associations. Third, the two-dimensional framework stems from the cultural theory of individualism and collectivism [1], yet our sample was relatively small and drawn from a single culture, which restricts the cross-cultural generalizability of our findings. Fourth, resting-state acquisitions characterize intrinsic organization; task-based self-construal priming would extend this work to situational changes. Fifth, the scatterplots, cluster-level sensitivity analyses, and sFC/FCV follow-ups use clusters selected in the same sample. Their effect sizes are therefore conditional on this selection [64], and the connectivity follow-ups and uncorrected brain–behavior correlations remain exploratory. Independent or cross-validated ROIs would strengthen these extensions. Finally, the reported analyses used r > 0.25 and a non-GSR pipeline; comparison with alternative thresholds and GSR pipelines would enable the assessment of generalization across preprocessing choices.

## 5. Conclusions

Using graph-theoretical analyses of resting-state fMRI data, the present study shows that independent and interdependent self-construal are associated with different patterns of voxelwise nodal centrality. At the primary nodal level, independent self-construal was positively associated with degree centrality in the left inferior occipital gyrus and right lingual gyrus. Interdependent self-construal was positively associated with degree centrality in the medial prefrontal cortex and thalamus. All four positive DC associations persisted in cluster-level sensitivity analyses adjusting for the other self-construal dimension and covariates. Exploratory sFC and FCV follow-ups characterize the distributed coupling of these hubs, linking perceptual and social-integrative organization to self-construal. Future cross-cultural and task-based studies can extend these associations across populations and situational contexts.

## Figures and Tables

**Figure 1:**
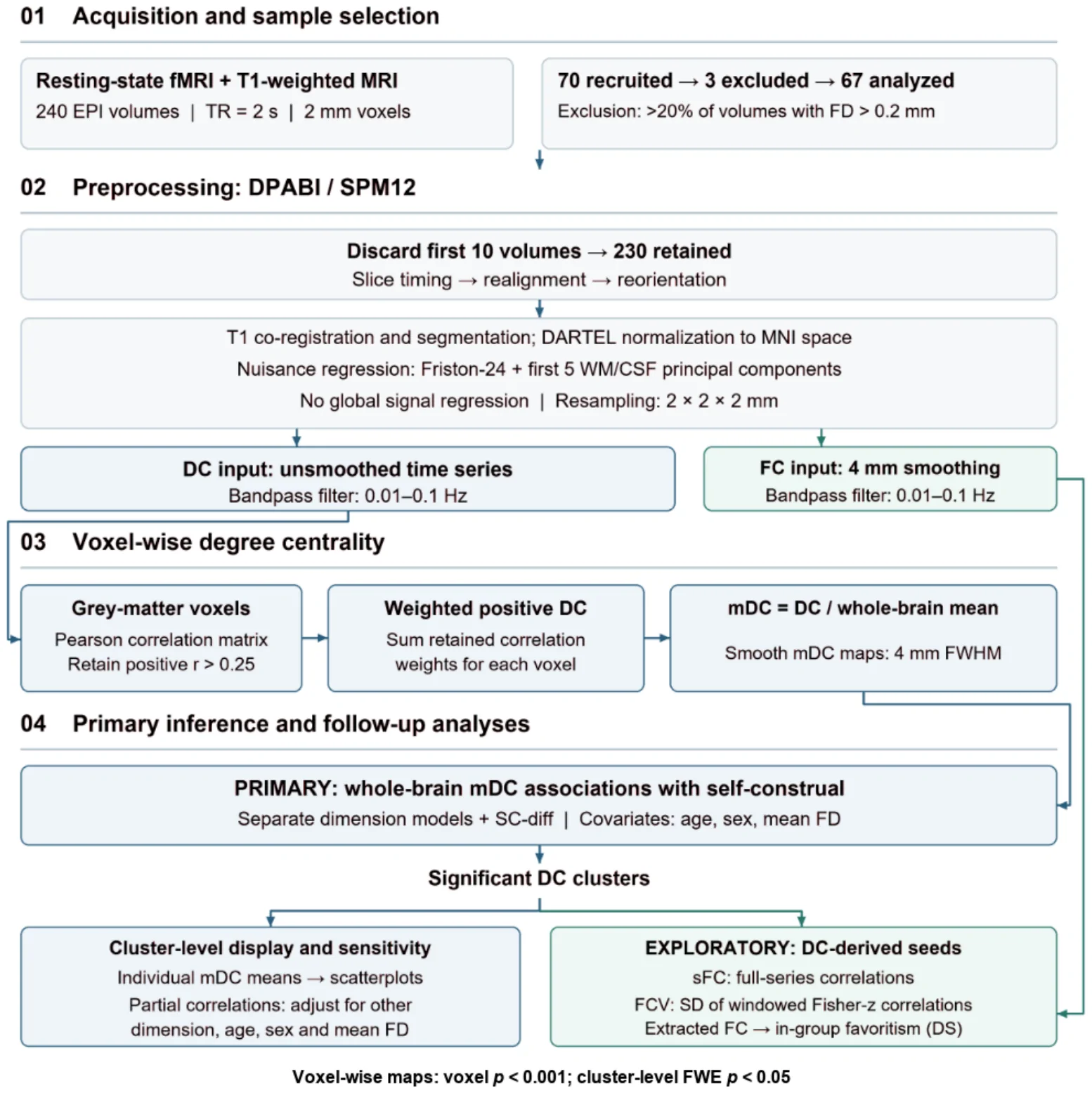
Workflow from data acquisition and preprocessing to primary degree centrality (DC) analysis and exploratory connectivity follow-ups. Arrows indicate the flow of data and DC-derived seed selection. The DC and functional connectivity (FC) branches use unsmoothed and smoothed time series, respectively; DC maps are smoothed after computation and mean normalization (mDC). The lower branches show individual-data scatterplots and cluster-level sensitivity analyses, alongside seed-based static FC (sFC), FC variability (FCV), and brain–behavior analyses. FD, framewise displacement; WM/CSF, white matter/cerebrospinal fluid; FWE, family-wise error.

**Figure 2:**
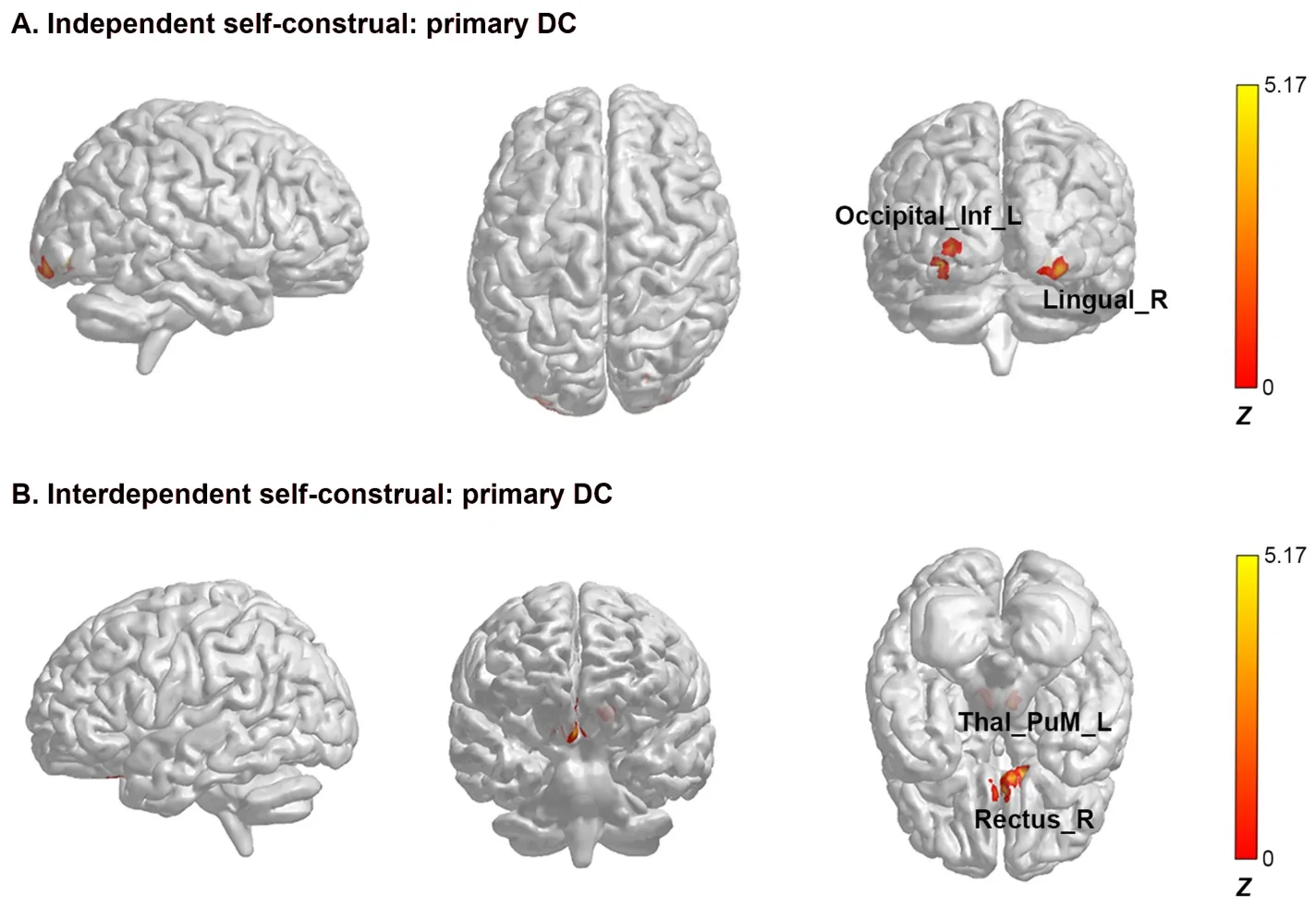
Primary DC analysis: brain regions where degree centrality (DC) was associated with self-construal, adjusting for age, sex and head motion (voxel-wise *p* < 0.001 with cluster-level FWE-corrected threshold of *p* < 0.05). (**A**) Clusters positively correlated with independent self-construal (left inferior occipital gyrus and right lingual gyrus). (**B**) Clusters positively correlated with interdependent self-construal (gyrus rectus/ventral MPFC and left medial pulvinar thalamus).

**Figure 3:**
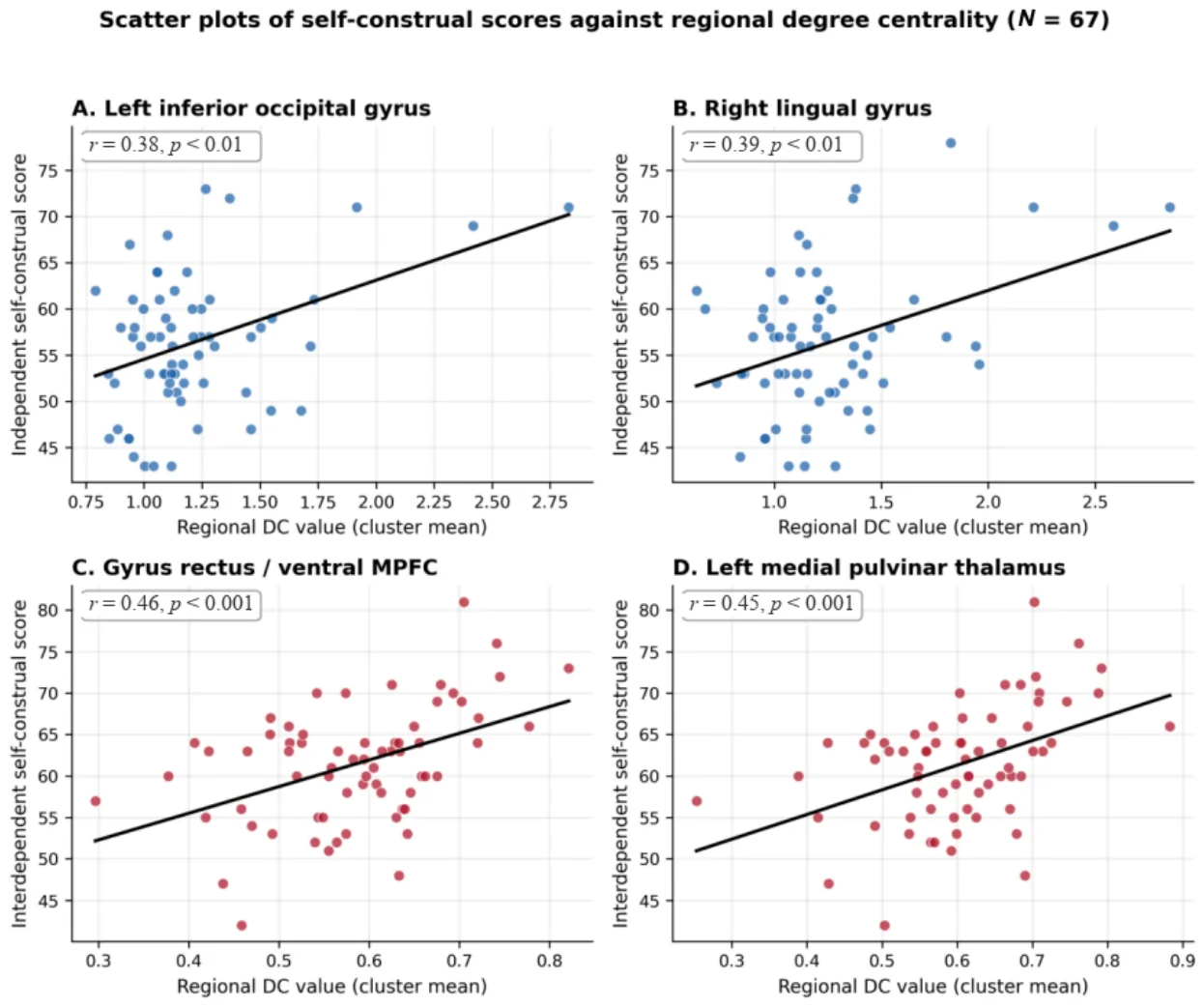
Scatter plots of self-construal scores against cluster-mean mDC values of the significant clusters (*N* = 67), with least-squares regression lines. (**A**) Independent self-construal and DC of the left inferior occipital gyrus cluster. (**B**) Independent self-construal and DC of the right lingual gyrus cluster. (**C**) Interdependent self-construal and DC of the gyrus rectus/ventral MPFC cluster. (**D**) Interdependent self-construal and DC of the left medial pulvinar thalamus cluster. Each point represents one participant. Reported r values are unadjusted Pearson correlations; the ROIs are the clusters identified in the primary DC analysis. Covariate-adjusted sensitivity results are reported in Section 3.2.

**Figure 4:**
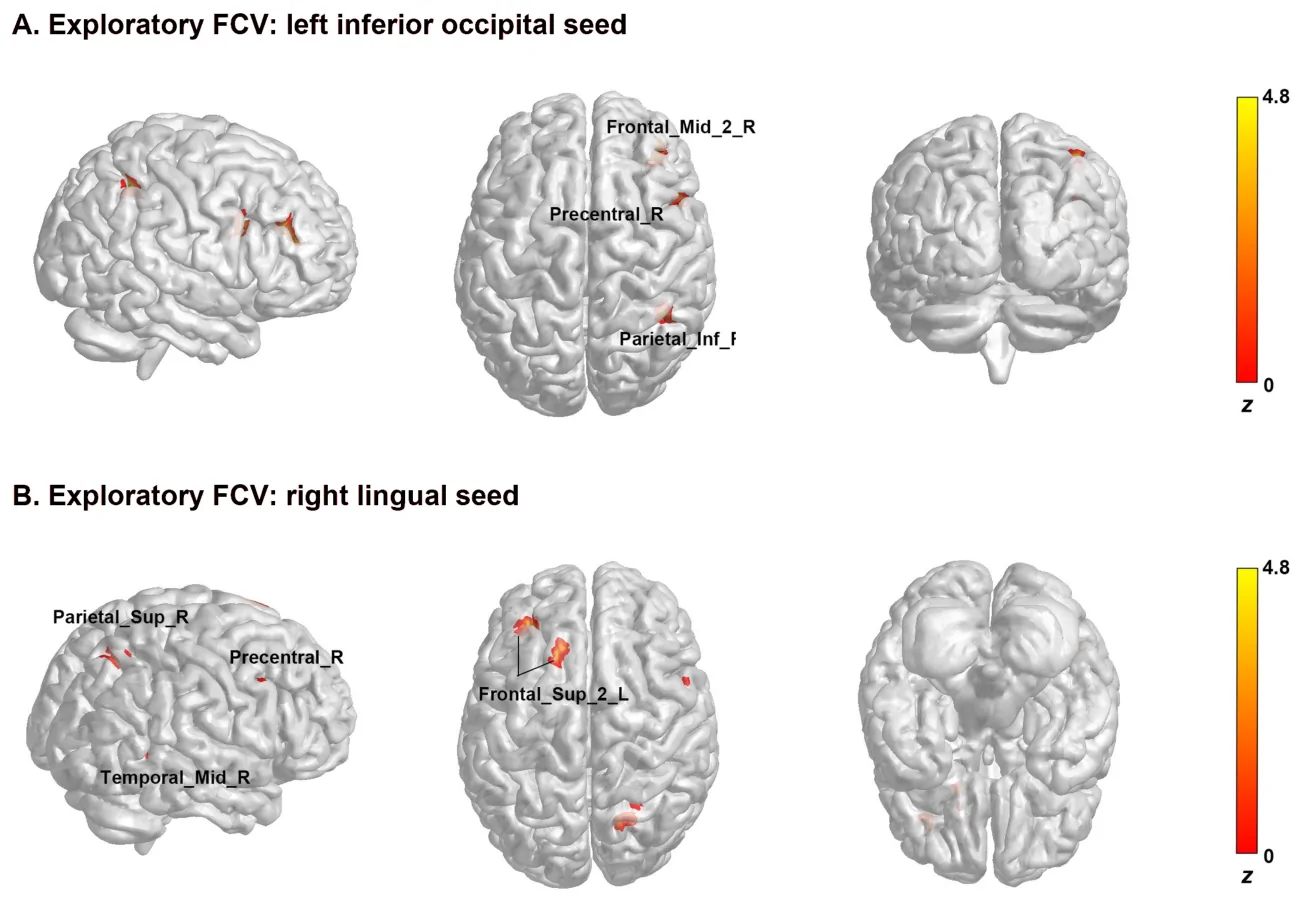
Exploratory functional connectivity variability (FCV) associated with independent self-construal, controlling for age, sex and head motion (voxel-wise *p* < 0.001, cluster-level FWE-corrected *p* < 0.05). (**A**) Clusters positively correlated with FCV between the left IOG seed and whole-brain voxels (right middle frontal gyrus, right precentral gyrus, and right inferior parietal lobule). (**B**) Clusters positively correlated with FCV between the right lingual seed and whole-brain voxels (left superior frontal gyrus, right precentral gyrus, right superior parietal lobule, and right middle temporal gyrus). No significant FCV clusters were found for interdependent self-construal at the same threshold.

**Table 1:** Primary analysis: brain regions where DC values were significantly related to independent and interdependent self-construal, adjusting for age, sex and head motion (voxel-wise *p* < 0.001, cluster-level FWE-corrected *p* < 0.05).

Dimension	Region (AAL)	k	Peak MNI (x, y, z)	Peak Z	*p* (Cluster, FWE)
Independent	Inferior occipital gyrus L (Occipital_Inf_L)	109	−22, −88, −6	4.61	<0.05
Independent	Lingual gyrus R (Lingual_R)	128	26, −84, −8	4.33	<0.05
Interdependent	Gyrus rectus/ventral MPFC (Rectus_R)	211	6, 18, −20	5.17	<0.05
Interdependent	Medial pulvinar thalamus L (Thal_PuM_L)	106	−14, −28, 12	4.74	<0.05

Note. All clusters exceeded the prespecified cluster-level FWE-corrected threshold of *p* < 0.05.

**Table 2:** Primary analysis: brain regions where DC values were significantly related to the self-construal difference score (SC-diff), adjusting for age, sex and head motion (voxel-wise *p* < 0.001, cluster-level FWE-corrected *p* < 0.05).

Contrast (SC-Diff)	Region (AAL)	k	Peak MNI (x, y, z)	Peak T	Peak Z	*p* (Cluster, FWE)
Positive	Gyrus rectus/ventral MPFC (Rectus_R)	90	6, 18, −20	4.31	4.02	0.016
Negative	Inferior occipital gyrus L (Occipital_Inf_L)	271	−22, −88, −4	5.61	5.03	<0.001
Negative	Lingual gyrus R (Lingual_R)	206	26, −84, −8	5.35	4.83	<0.001

**Table 3:** Exploratory static functional connectivity (seed-based) significantly related to independent and interdependent self-construal, controlling for age, sex and head motion (voxel-wise *p* < 0.001, cluster-level FWE-corrected *p* < 0.05). Independent-self-construal rows use Occipital_Inf_L and Lingual_R seeds; interdependent-self-construal rows use Rectus_R and Thal_PuM_L seeds.

Seed	Region (AAL)	k	Peak MNI (x, y, z)	Peak Z	*p* (Cluster, FWE)
Occipital_Inf_L	Posterior orbital frontal cortex L (OFCpost_L)	136	−38, 18, −16	4.92	<0.05
Occipital_Inf_L	Postcentral gyrus L (Postcentral_L)	108	−32, −42, 68	4.18	<0.05
Occipital_Inf_L	Middle temporal gyrus L (Temporal_Mid_L)	96	−52, −22, −10	4.12	<0.05
Occipital_Inf_L	Middle occipital gyrus L (Occipital_Mid_L)	104	−38, −74, 22	4.30	<0.05
Lingual_R	Posterior orbital frontal cortex L (OFCpost_L)	222	−40, 24, −18	5.02	<0.05
Lingual_R	Posterior orbital frontal cortex R (OFCpost_R)	81	44, 30, −14	4.24	<0.05
Lingual_R	Superior medial frontal gyrus L (Frontal_Sup_Medial_L)	108	−4, 42, 44	3.99	<0.05
Lingual_R	Superior medial frontal gyrus R (Frontal_Sup_Medial_R)	174	8, 54, 32	4.06	<0.05
Lingual_R	Superior medial frontal gyrus R (Frontal_Sup_Medial_R)	108	4, 64, 12	5.11	<0.05
Lingual_R	Postcentral gyrus L (Postcentral_L)	114	−40, −42, 60	4.00	<0.05
Lingual_R	Middle temporal gyrus L (Temporal_Mid_L)	203	−52, −22, −12	4.78	<0.05
Lingual_R	Middle temporal gyrus L (Temporal_Mid_L)	119	−58, 0, −30	4.24	<0.05
Lingual_R	Middle temporal gyrus R (Temporal_Mid_R)	121	52, −2, −26	4.35	<0.05
Lingual_R	Parahippocampal gyrus R (ParaHippocampal_R)	80	24, −2, −32	5.27	<0.05
Lingual_R	Fusiform gyrus L (Fusiform_L)	121	−31, −36, −25	4.77	<0.05
Lingual_R	Angular gyrus L (Angular_L)	104	−55, −56, 23	3.79	<0.05
Lingual_R	Middle occipital gyrus L (Occipital_Mid_L)	227	−46, −80, 18	4.10	<0.05
Rectus_R	Lingual gyrus L (Lingual_L)	122	−14, −94, −12	4.41	<0.05
Rectus_R	Calcarine cortex R (Calcarine_R)	102	8, −82, 0	4.02	<0.05
Thal_PuM_L	Inferior temporal gyrus L (Temporal_Inf_L)	88	−46, −32, −22	4.53	<0.05
Thal_PuM_L	Calcarine cortex L (Calcarine_L)	404	−6, −88, −14	4.56	<0.05
Thal_PuM_L	Middle occipital gyrus L (Occipital_Mid_L)	225	−26, −68, 38	4.55	<0.05
Thal_PuM_L	Fusiform gyrus R (Fusiform_R)	110	32, −78, −12	3.97	<0.05
Thal_PuM_L	Insula L (Insula_L)	71	−40, −2, 0	4.86	<0.05

Note. All clusters exceeded the prespecified cluster-level FWE-corrected threshold of *p* < 0.05.

**Table 4:** Exploratory static functional connectivity (seed-based) significantly related to the self-construal difference score (SC-diff), controlling for age, sex and head motion (voxel-wise *p* < 0.001, cluster-level FWE-corrected *p* < 0.05). All reported SC-diff associations in this table are negative.

Seed	Region (AAL)	k	Peak MNI (x, y, z)	Peak T	Peak Z	*p* (Cluster, FWE)
Occipital_Inf_L	Lingual_R	459	10, −90, −10	5.21	4.73	<0.001
Occipital_Inf_L	Postcentral_L/parietal	441	−16, −30, 74	4.36	4.05	<0.001
Lingual_R	Lingual_L/bilateral occipital	1600	−18, −66, −12	4.73	4.35	<0.001
Lingual_R	Lingual_R (anterior)	312	8, −56, −4	5.63	5.04	<0.001
Lingual_R	Calcarine_R	113	20, −82, 16	4.77	4.39	0.002
Lingual_R	Frontal_Sup_2_L	68	−16, 64, 8	4.79	4.40	0.038
Lingual_R	Postcentral_L	491	−20, −34, 54	5.09	4.64	<0.001
Lingual_R	Postcentral_R	150	14, −36, 64	5.30	4.80	<0.001
Lingual_R	Precuneus_R	84	6, −34, 58	4.35	4.05	0.013

**Table 5:** Exploratory functional connectivity variability (FCV) associated with independent self-construal, controlling for age, sex and head motion (voxel-wise *p* < 0.001, cluster-level FWE-corrected *p* < 0.05).

Seed	Region (AAL)	k	Peak MNI (x, y, z)	Peak Z	*p* (Cluster, FWE)
Occipital_Inf_L	Middle frontal gyrus R (Frontal_Mid_2_R)	89	38, 36, 32	4.57	<0.05
Occipital_Inf_L	Precentral gyrus R (Precentral_R)	101	52, 12, 36	4.34	<0.05
Occipital_Inf_L	Inferior parietal lobule R (Parietal_Inf_R)	40	44, −48, 54	4.26	<0.05
Lingual_R	Superior frontal gyrus L (Frontal_Sup_2_L)	35	−28, 38, 36	4.72	<0.05
Lingual_R	Superior frontal gyrus L (Frontal_Sup_2_L)	31	−16, 22, 62	4.27	<0.05
Lingual_R	Precentral gyrus R (Precentral_R)	30	52, 6, 36	4.46	<0.05
Lingual_R	Superior parietal lobule R (Parietal_Sup_R)	48	20, −66, 48	4.17	<0.05
Lingual_R	Middle temporal gyrus R (Temporal_Mid_R)	26	50, −56, 2	4.21	<0.05

Note. All clusters exceeded the prespecified cluster-level FWE-corrected threshold of *p* < 0.05.

## Data Availability

The data that support the findings of this study are only available from the corresponding author upon reasonable request due to containing information that could compromise the privacy of research participants.
